# GSK3β N-terminus binding to p53 promotes its acetylation

**DOI:** 10.1186/1476-4598-8-14

**Published:** 2009-03-05

**Authors:** Tae-Yeon Eom, Richard S Jope

**Affiliations:** 1 Department of Cell Biology, University of Alabama at Birmingham, Birmingham, Alabama 35294, USA; 2 Department of Psychiatry and Behavioral Neurobiology, University of Alabama at Birmingham, Birmingham, Alabama 35294, USA

## Abstract

The prevalence in human cancers of mutations in p53 exemplifies its crucial role as a tumor suppressor transcription factor. Previous studies have shown that the constitutively active serine/threonine kinase glycogen synthase kinase-3β (GSK3β) associates with the C-terminal basic domain of p53 and regulates its actions. In this study we identified the GSK3β N-terminal amino acids 78–92 as necessary for its association with p53. Inhibitors of GSK3 impaired the acetylation of p53 at Lys373 and Lys382 near the GSK3β binding region in p53, indicating that GSK3β facilitates p53 acetylation. We also found that acetylation of p53 reduced its association with GSK3β, as well as with GSK3α. These results indicate that the N-terminal region of GSK3β binds p53, this association promotes the acetylation of p53, and subsequently acetylated p53 dissociates from GSK3.

## Findings

Glycogen synthase kinase-3 (GSK3) phosphorylates more than 40 substrates, so its actions must be controlled in a substrate-specific manner to avoid spurious phosphorylation of unintended substrates upon fluxes in the activity of GSK3 [1]. This must be accomplished by synchronous regulation of GSK3 binding to substrates or substrate-containing protein complexes and regulation of GSK3 activity, such as the activity-regulating serine-phosphorylation of GSK3. Thus, the association of GSK3 in protein complexes is likely as critical as post-translational modifications in controlling the actions of GSK3. This has been well-described for the Wnt signaling pathway where GSK3 must be bound to axin to phosphorylate axin-bound β-catenin [2]. This substrate specificity implies the existence of different GSK3 recognition motifs for various binding partners, but GSK3 binding domain studies have been confined to Wnt signaling proteins [3-5]. Thus, little is known about binding domains in GSK3, and it is usually depicted as three domains, a small N-terminal domain, a slightly larger C-terminal domain, and a predominant middle kinase domain. Additionally, a nuclear localization sequence was recently identified [6].

To understand better the protein-protein interactions of GSK3β, we investigated the residues required for GSK3β to bind the tumor suppressor p53 [7]. GSK3β forms a complex with nuclear p53 to promote p53-induced apoptosis, and the C-terminal p53 basic domain is necessary for this interaction [8-10]. GSK3β also interacts with p53 in the nucleus during cellular senescence [11], and GSK3β binds p53 in mitochondria [9]. Although the interaction between GSK3β and p53 has been confirmed in several studies, the functional consequences are controversial, possibly because of the many other regulatory influences on p53 and the context- and cell-specific regulation and actions of p53. GSK3 has been reported to phosphorylate Ser33-p53 [12] or Ser315-p53 and Ser376-p53 [13,14], and to regulate the intracellular localization of p53 [10,13,14]. In this study we identified the domain of GSK3β necessary for its association with p53. Furthermore, we found that GSK3 promotes the acetylation of p53, and that p53 acetylation reduces its association with GSK3β.

**Figure 1:**
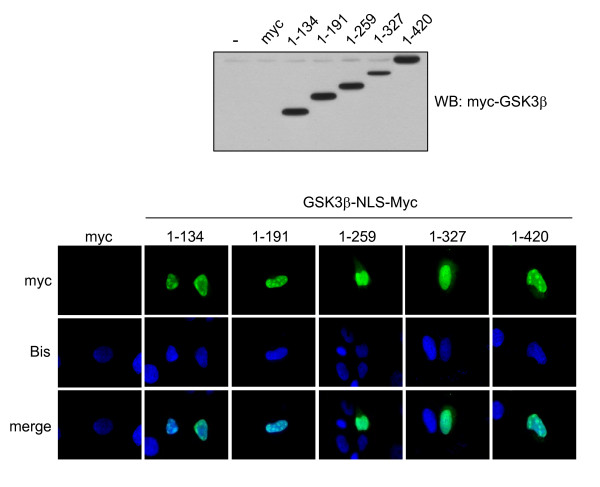
**Nuclear localization of expressed GSK3β constructs**. p53-null human lung carcinoma H1299 cells that express inducible wild-type HA-tagged p53 were transiently transfected with wild-type GSK3β-NLS-myc (1–420) or the indicated mutants of GSK3β-NLS-myc. Constructs generated by ligating rat GSK3β cDNA into the pShooter vector pCMV/myc/nuc (Invitrogen) were expressed using FuGENE 6 (Roche). NLS-myc vector (myc) was used as a negative control. After 24 hr, expression was examined by immunoblotting and immunostaining with anti-myc-tag (Cell Signaling) and Alexa Fluor 488 goat anti-mouse IgG (Invitrogen). Nuclei were labeled with 1 μg/ml bisbenzimide (Bis; blue). The expression level of each mutant construct was similar to wild-type GSK3β-NLS-myc and all GSK3β-NLS-myc constructs (green) were expressed in the nucleus. 100× magnification.

**Figure 2:**
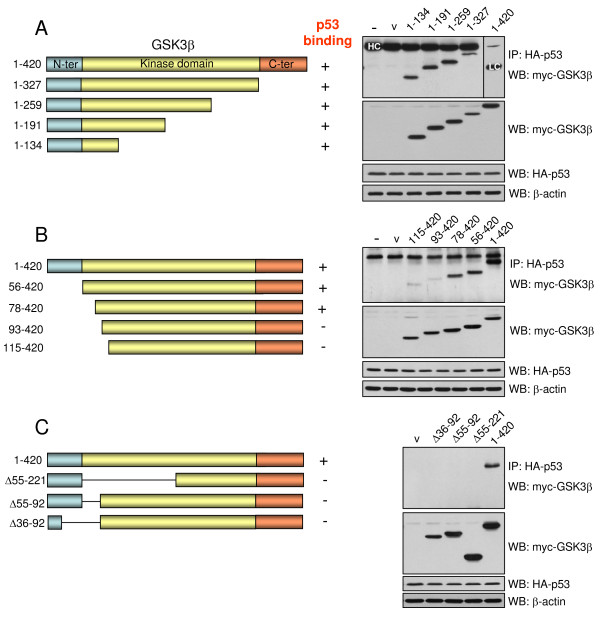
**Identification of the GSK3β domain required for association with p53**. Schematic diagrams (left) depict GSK3β constructs (A) sequentially truncated from the C-terminal, (B) sequentially truncated from the N-terminal, and (C) deletion constructs. GSK3β constructs were transiently transfected into H1299 cells and HA-p53 was inducibly expressed by removing doxycycline from the medium for 24 hr [9]. Cells were subjected to lysis, immunoprecipitation using sheep anti-mouse IgG Dynabeads (Dynal Biotech) and 1 μg anti-HA (Covance) and immunoblotting as indicated. The heavy chain (HC) and light chain (LC) of IgG are indicated, and β-actin was used as loading control.

The region of GSK3β that binds to p53 was examined by expressing mutants of myc-tagged GSK3β fused to a nuclear localization sequence (NLS) in p53-null H1299 cells that inducibly express wild-type HA-tagged p53. Immunostaining of transfected cells demonstrated that all GSK3β constructs were expressed in the nucleus (Figure 1 and data not shown). Following expression of NLS-GSK3β constructs, the expression of HA-p53 was induced, and co-immunoprecipitation of p53 with GSK3β constructs was measured. GSK3β was sequentially truncated from the C-terminal to the smallest construct consisting of residues 1–134, and each of these GSK3β constructs associated with p53 (Figure 2A), indicating that the N-terminal amino acids 1–134 of GSK3β are required for binding to p53. Subsequently, smaller 25-residue N-terminal sequential truncations of GSK3β were expressed and these showed that deletion of the N-terminal 77 residues did not abrogate binding to p53, but deletion of the N-terminal 92 or 114 residues eliminated association with p53 (Figure 2B). This demonstrates that a region encompassing residues 78–92 of GSK3β was necessary for p53 binding. This localization was confirmed by constructing three different deletion mutants of GSK3β with this region eliminated, which failed to bind p53 (Figure 2C).

**Figure 3:**
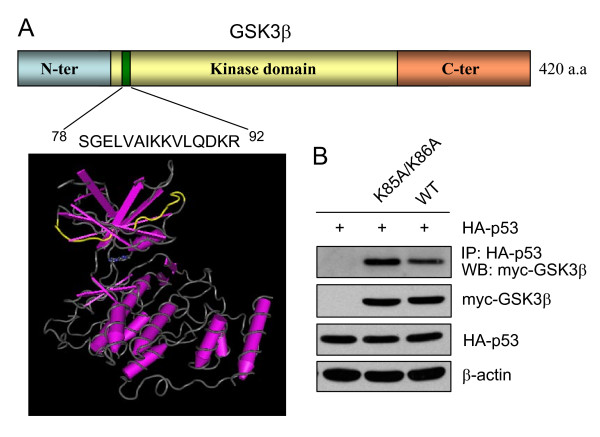
**Diagram of the GSK3β region required for association with p53**. (A) The sequence of GSK3β shows the region required for association with p53, consisting of amino acids 78–92, that is located close to the N-terminus of GSK3β. The molecular rendering of a monomer of GSK3β was generated using the Cn3D software from the NCBI based on the protein data bank file 1O9U , and the p53 binding domain of GSK3β was depicted as yellow. (B) H1299 cells were transiently transfected to express wild-type GSK3β-NLS-myc (1–420 amino acids; WT) or kinase-dead GSK3β-NLS-myc (K85A/K86A), and HA-p53 was inducibly expressed, for 24 hr. Cells were subjected to lysis and anti-HA was used to immunoprecipitate p53, followed by immunoblotting with anti-myc antibody to detect GSK3β-NLS-myc. The levels of expressed HA-p53, NLS-myc-GSK3β, and β-actin were determined by immunoblotting.

Residues 78–92 of GSK3β that are required for association with p53 reside close to the N-terminus of GSK3β within the β-strand (Figure 3A). This region of GSK3β required for association with p53 is composed of two externally exposed loops that might be easily assessable to binding partners and contains K85/K86 that are critical for GSK3β kinase activity. Thus, we tested if GSK3β activity is required for its association with p53. Kinase dead GSK3β was generated by mutating K85 and K86 to alanine, with an NLS and myc-tag (K85A/K86A-NLS-GSK3β). Co-immunoprecipitation showed that both wild-type and kinase-dead GSK3β associate with p53, demonstrating that GSK3β activity is not required for association with p53 (Figure 3B).

**Figure 4:**
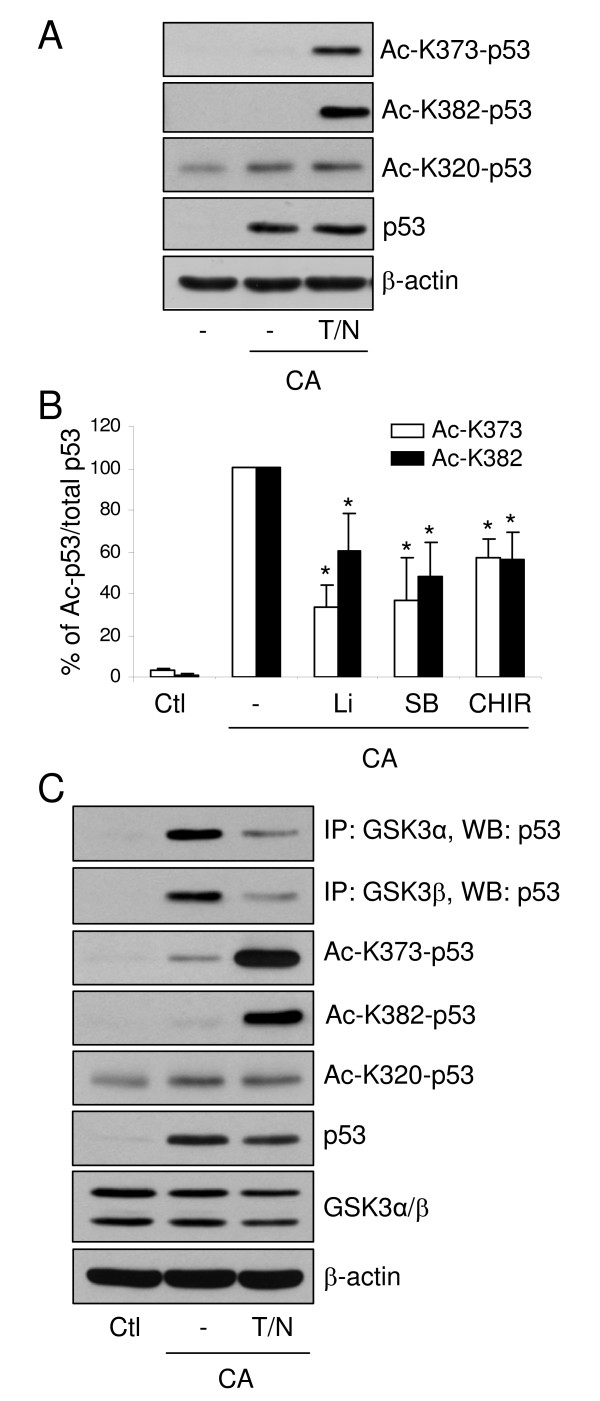
**GSK3 regulates p53 acetylation which regulates association with GSK3β**. (A) SH-SY5Y cells were preincubated with 1 μM TSA and 5 mM nicotinamide (T/N) for 2 hr and treated with 1 μM camptothecin (CA) for 3 hr. Total p53 was immunoprecipitated followed by immunoblotting with antibodies specific for acetylated Lys373 (Ac-K373-p53), Lys382 (Ac-K382-p53), Lys320 (Ac-K320-p53) (Trevigen), total p53 (Santa Cruz), and β-actin (Sigma). (B) Quantitative analysis of the percentage of acetylated p53. SH-SY5Y cells were preincubated with 1 μM TSA and 5 mM nicotinamide for 2 hr, with GSK3 inhibitors, 20 mM lithium (Li), 10 μM SB216763 (SB), or 10 μM CHIR99021 (CHIR), for 30 min, and treated with 1 μM camptothecin for 3 hr. Quantitative values are means ± S.E.; n = 3. *p < 0.05, ANOVA with Dunnett's post hoc multiple comparisons test. (C) SH-SY5Y cells were preincubated with 1 μM TSA and 5 mM nicotinamide (T/N) for 2 hr and treated with 1 μM camptothecin (CA) for 3 hr. The association of GSK3α/β with p53 was assessed by immunoprecipitation of GSK3α or GSK3β followed by immunoblotting total p53. The endogenous total p53, total GSK3α/β, acetylated p53 (Ac-K373-p53, Ac-K382-p53, Ac-K320-p53), and β-actin levels were determined by immunoblotting.

We examined if GSK3 regulates p53 acetylation because GSK3β binds p53 C-terminal 364–373 residues [9] containing three sites of p53 acetylation (K370, K372, K373) and very close to two other acetylation sites (K381, K382). Previously, the coactivators p300 and PCAF were shown to acetylate the C-terminus region of p53, which activates the DNA binding activity of p53 [15,16]. In addition to this role, the acetylation of p53 contributes to regulating its stability, transcriptional activity, and localization [17-19]. Trichostatin A (TSA) is a well-established inhibitor of histone deacetylases (HDACs) in classes I, II, and IV, comprising HDAC 1–11, and nicotinamide inhibits HDAC class III, comprising Sirt1-7 [20]. Therefore, SH-SY5Y cells were treated with 1 μM camptothecin for 3 hr to increase p53 levels, with or without 1 μM TSA plus 5 mM nicotinamide to inhibit p53 deacetylation. p53 levels were increased by camptothecin treatment and co-treatment with TSA/nicotinamide increased p53 acetylation at K373 and K382, but not at K320 (Figure 4A). Treatment with three structurally diverse GSK3 inhibitors, lithium, SB216763, and CHIR99021, significantly inhibited the acetylation of p53 at K373 and K382 (Figure 4B). In contrast, lithium and SB216763 did not inhibit p53 K320 acetylation (99 ± 5% and 140 ± 15%, respectively). These results implicate GSK3 as a modulator of p53 acetylation, indicating that GSK3 promotes p53 acetylation.

We investigated if acetylation of p53 regulates its association with GSK3 since p53 acetylation sites are within, or close to, the residues of p53 required for its association with GSK3. SH-SY5Y cells were treated with 1 μM camptothecin for 3 hr to increase p53 levels, in the absence or presence of 1 μM TSA plus 5 mM nicotinamide to inhibit the deacetylation of p53. Each isoform of GSK3 was immunoprecipitated and p53 was found to co-immunoprecipitate with both GSK3 isoforms. Inclusion of TSA/nicotinamide with camptothecin treatment increased p53 acetylation on K373 and K382 but not on K320, and there was a large inhibition of the association of p53 with both GSK3 isoforms compared with their association with p53 in the absence of TSA/nicotinamide (Figure 4C). These results indicate that acetylation of p53 at K373 and K382, proximal to the GSK3β binding region, inhibits the association of GSK3 with p53.

p53 and GSK3 each has critical roles in determining cell survival [1,7]. Therefore, it is particularly interesting to determine mechanisms regulating interactions between the two proteins. In this study we identified a novel protein-interacting region near the N-terminus of GSK3 that is critical for its interaction with p53. Furthermore, we found that GSK3 promotes the acetylation of p53 at sites (K373 and K382) near the GSK3β-binding region of p53, which spans residues 364–373. Highly acetylated p53, in turn only poorly associates with GSK3. These findings indicate that a region near the N-terminus of GSK3 associates with the C-terminal basic domain of p53 that contains several sites that can be acetylated, GSK3 promotes the acetylation of p53, and acetylated p53 dissociates from GSK3.

Little is known about domains in GSK3 that are critical for its interactions with other proteins despite the importance of these interactions for controlling and directing the actions of GSK3 [1]. Since the interaction between GSK3β and p53 is important for controlling the actions of both proteins [8], we examined regions in GSK3 that are required for this interaction. Previously overlapping but non-identical binding sites in the carboxy lobe of GSK3β were identified as critical for associating with proteins in the Wnt signaling pathway, axin and GBP/FRAT [3-5]. Both axin and GBP bind a channel in GSK3β formed by an α-helix (residues 262–273) and an extended loop (residues 285–299), so their binding is mutually exclusive, but the interacting residues of GSK3β differ for axin and GBP [2]. Unlike the Wnt signaling proteins, this entire C-terminal region of GSK3 was completely dispensable for its interaction with p53, which required GSK3β residues 78–92. Although the binding domain contains residues K85/K86 that are necessary for its kinase activity, kinase-dead GSK3β with these residues mutated to alanine associated with p53 equivalently to wild-type GSK3β indicating that GSK3β activity does not affect the interaction with p53, which has also been shown for kinase-dead GSK3β binding to components of the Wnt signaling system [21]. Thus, these results demonstrate that different domains of GSK3β are utilized to enable GSK3β to regulate the Wnt and p53 systems. Differential protein-associating domains of GSK3β likely contribute to enabling GSK3 to selectively regulate phosphorylation of its substrates, which number over forty.

Several effects of GSK3 on p53 have been reported, including facilitation of its transcriptional activity and apoptosis [8-10], p53 phosphorylation [12-14], and p53 intracellular localization and trafficking [22]. Since the region encompassing residues 364–373 in the C-terminal basic domain of p53 that is required for its association with GSK3β contains several sites that can be acetylated, we examined if GSK3 regulates p53 acetylation and if p53 acetylation regulates its association with GSK3. Both of these were found to occur, as GSK3 inhibitors substantially reduced the acetylation of p53 at two sites near the region of p53 required for its association with GSK3β, residues K373 and K382, but not at a more distant acetylation site, K320, and increased acetylation of p53 decreased its association with GSK3. Although the functional roles of p53 acetylation remain controversial, several reports suggest that acetylation increases p53 stability and promotes co-activator recruitment, leading to transcriptional activation of target genes [23]. Our findings indicate that the N-terminal region of GSK3 associates with the basic domain of p53 and this facilitates p53 acetylation which leads to dissociation of p53 from GSK3.

## Competing interests

The authors declare that they have no competing interests.

## Authors' contributions

TYE performed the research and RSJ supervised the study and drafted the manuscript.
